# Identification of miRNA–mRNA Pairs in the Alzheimer’s Disease Expression Profile and Explore the Effect of miR-26a-5p/PTGS2 on Amyloid-β Induced Neurotoxicity in Alzheimer’s Disease Cell Model

**DOI:** 10.3389/fnagi.2022.909222

**Published:** 2022-06-15

**Authors:** Tao Xie, Yongyan Pei, Peijia Shan, Qianqian Xiao, Fei Zhou, Liuqing Huang, Shi Wang

**Affiliations:** ^1^Department of Neurology, The Third Affiliated Hospital of Naval Medical University, Shanghai, China; ^2^School of Chemistry and Chemical Engineering, Guangdong Pharmaceutical University, Zhongshan, China; ^3^Department of Neurology, Changhai Hospital, Naval Medical University, Shanghai, China

**Keywords:** Alzheimer’s disease, miRNA–mRNA, miR-26a-5p/PTGS2, neurotoxicity, baicalein

## Abstract

Alzheimer’s disease (AD) is a progressive neurodegenerative disease and the most common type of dementia. MicroRNAs (miRNAs) have been extensively studied in many diseases, including AD. To identify the AD-specific differentially expressed miRNAs and mRNAs, we used bioinformatics analysis to study candidate miRNA–mRNA pairs involved in the pathogenesis of AD. These miRNA–mRNAs may serve as promising biomarkers for early diagnosis or targeted therapy of AD patients. In this study, based on the AD mRNA and miRNA expression profile data in Gene Expression Omnibus (GEO), through differential expression analysis, functional annotation and enrichment analysis, weighted gene co-expression network analysis, miRNA–mRNA regulatory network, protein–protein interaction network, receiver operator characteristic and Least absolute shrinkage and selection operator (LASSO) regression and other analysis, we screened the key miRNA–mRNA in the progress of AD: miR-26a-5p/PTGS2. Dual-luciferase and qPCR experiments confirmed that PTGS2 is a direct target gene of miR-26a-5p. The expression of miR-26a-5p in the peripheral blood of AD patients and AD model cells (SH-SY5Y cells treated with Aβ_25–35_) was up-regulated, and the expression of PTGS2 was down-regulated. Functional gain -loss experiments confirmed that PTGS2 protects AD model cells from damage by inhibiting proliferation and migration. However, the expression of miR-26a-5p promotes the proliferation of AD model cells. It is further found that PTGS2 is involved in the regulation of miR-26a-5p and can reverse the effect of miR-26a-5p on the proliferation of AD model cells. In addition, through network pharmacology, qPCR and CCK-8, we found that baicalein may affect the progression of AD by regulating the expression of PTGS2. Therefore, PTGS2 can be used as a target for AD research, and miR-26a-5p/PTGS2 can be used as an axis of action to study the pathogenesis of AD.

## Introduction

Alzheimer’s disease (AD) is a progressive neurodegenerative disease characterized by progressive cognitive impairment. The main clinical manifestations are memory loss and behavior changes, especially in elderly patients (Tiwari et al., 2019; Breijyeh and Karaman, 2020). Amyloid beta (Aβ) plaques and neurofibrillary tangles are key neuropathological features of AD, but their pathogenesis is complex, including neuronal dysplasia, nerve injury and release of neuroinflammatory mediators, resulting in the difficulty of early diagnosis of AD (Congdon and Sigurdsson, 2018; Busche and Hyman, 2020; Shigemizu et al., 2020). It also hinders the development of effective therapeutic strategies to delay or prevent the development of AD (Tiwari et al., 2019). Therefore, the identification of AD-related molecules is of great significance for the development of new methods for the diagnosis and treatment of AD.

Omics methods based on microarray and high-throughput sequencing techniques have been used to investigate the pathological mechanisms of AD (Noh et al., 2014). Many sequencing data are used to build open databases for various diseases, such as The Cancer Genome Atlas (TCGA) and Gene Expression Omnibus (GEO) (Barrett et al., 2013; Hutter and Zenklusen, 2018). Integrating these RNA sequencing data to identify functional drivers of diseases and predict their specific regulatory functions has become an efficient research method. Currently, many researches focus on predicting the function of target genes or miRNAs. MicroRNAs (miRNAs) are a group of small non-coding RNAs whose abnormal expression has been found in the disease progression of various human diseases (Ali Syeda et al., 2020; Serpente et al., 2020; Yoshida et al., 2021). miRNAs play an important regulatory role in gene expression and various cellular processes such as differentiation and apoptosis mainly by directly binding to the 3′UTR of the target gene (Di Leva et al., 2014). More and more studies have shown that miRNAs play a key role in the pathogenesis of AD (Serpente et al., 2020). For example, Tao et al. (2021) reported that miR-204-3p targeting Nox4 mediates memory deficits in a mouse model of AD. Hou et al. (2020) found that correcting the abnormal signal of miR-124/PTPN1 can save the pathology of tau in AD, which implies the key role of miR-124/PTPN1 in the progression of AD. Ansari et al. (2019) provided evidence of miR-146a and miR-181a as biomarkers for AD diagnosis. Therefore, the identification of new miRNAs/mRNA can provide very important data reference for the study of pathogenesis, diagnosis and therapeutic target screening of AD.

In recent years, various cellular models have been developed to better understand the pathogenesis of AD. Amyloid β accumulation is one of the characteristics of AD, and Aβ_25–35_ is one of the toxic forms of AD, which has been widely used in *in vitro* model induction. Cai et al. (2018) established an AD cell model by adding Aβ_25–35_ to SH-SY5Y cells, and they found that apoptosis may play a role in AD-related neuronal loss. Similarly, the human neuroblastoma cell line SH-SY5Y is widely used in the study of neurodegenerative diseases such as Parkinson’s disease and AD (de Medeiros et al., 2019; Ramalingam et al., 2019).

In this study, we aim to identify differentially expressed miRNAs and mRNAs from the Comprehensive Gene Expression Database (GEO) and then use weighted gene co-expression network analysis (WGCNA), miRNA-mRNA regulatory network, protein–protein interaction (PPI) network, receiver operator characteristic (ROC) and least absolute shrinkage and selection operator (LASSO) Regression, Correlation analysis, qPCR and dual-luciferase analysis methods to explore possible miRNA-mRNA association pairs between differentially expressed miRNAs and mRNA. These miRNAs and mRNA may be the basis of the molecular mechanism of AD. Subsequently, *in vitro* cell experiments were used to verify the impact of key miRNA-mRNA associations on AD model cells. Finally, based on network pharmacology and *in vitro* experiments, the main Chinese herbal medicine components related to key mRNA are analyzed.

## Materials and Methods

### Data Collection

The mRNA (GSE140829, GSE97760, GSE85426) and miRNA (GSE120584, GSE157239, GSE46579) expression profiles of AD are downloaded from Gene expression Omnibus (GEO^1^). The detailed information of all downloaded data sets is summarized in Table 1.

**TABLE 1:** Information of GEO datasets.

Dataset	Platform		AD	Normal	Sample type
GSE140829	GPL15988	HumanHT-12 v4 Expression BeadChip (nuID)	204	249	Peripheral blood
GSE97760	GPL16699	Agilent-039494 SurePrint G3 Human GE v2 8 × 60K Microarray 039381	9	10	Peripheral blood
GSE85426	GPL14550	Agilent-028004 SurePrint G3 Human GE 8 × 60K Microarray	90	90	Peripheral blood
GSE120584	GPL21263	3D-Gene Human miRNA V21_1.0.0	1021	288	Blood serum
GSE157239	GPL21572	Affymetrix Multispecies miRNA-4 Array [ProbeSet ID version]	8	8	Tissue
GSE46579	GPL11154	Illumina HiSeq 2000 (Homo sapiens)	48	22	Peripheral blood

*AD, Alzheimer’s disease. Normal, non-dementia controls.*

### Data Processing of Differential Expression mRNAs and miRNAs

First, we use the SVA package in sangerbox^2^ to merge GSE140829 and GSE97760 and remove batch effects. Then, the limma software package was used to analyze the differentially expressed RNAs between the AD group and the control group. The screening criteria for differentially expressed genes and miRNAs are: | log2 Fold Change (FC)| > 1, False Discovery Rates (FDR) < 0.05 and *p* < 0.05. Finally, the differentially expressed miRNAs and miRNAs screened were drawn as heat maps and Volcano plot by Sangerbox.

### Weighted Gene Co-expression Network Analysis

Use the WGCNA package in sangerbox to analyze the GSE157239 miRNA expression data. First, the system determines the appropriate β Soft threshold, and finally select the best soft threshold β = 4 conduct follow-up analysis. Then hierarchically cluster according to the miRNA expression value, and draw a dynamic tree to identify different modules. Divide the miRNAs with high similarity into the same module. Then calculate the module eigengene (ME) value of each module. And calculate the correlation coefficient between ME value and phenotype.

### Construction of miRNA–mRNA Regulatory Network

In order to explore the correlation between miRNA and mRNA and analyze the potential role of these RNAs in AD, we constructed a miRNA-mRNA regulatory network. First, miRWalk^3^ and Starbase^4^ are used to predict the target mRNA of differentially expressed miRNA. Then compare the target mRNA with the differentially expressed mRNAs to obtain the key mRNAs. Use miRNA–mRNA interaction to construct miRNA-mRNA regulatory network, and use Cytoscape 3.9.0 to visualize.

### Functional Enrichment Analysis

Import mRNAs from the miRNA-mRNA regulatory network into the Database for Annotation, Visualization and Integrated Discovery (DAVID 6.8^5^) for Gene Ontology (GO) and Kyoto Encyclopedia of Genes and Genomes (KEGG) analyze. The enrichment analysis of GO includes biological process (BP), cell component (CC) and molecular function (MF). *P* < 0.05 was considered statistically significant. The results are visualized with sangerbox.

### Protein–Protein Interaction Network Analysis

Use STRING 11.5^6^ to analyze the functional connection and interaction of the encoded gene proteins in miRNA-mRNAs. Keep interaction pairs with confidence ≥ 0.4. Then use Cytoscape 3.9.0 to construct a PPI network. Subsequently, the Cytoscape plug-in Molecular Complex Detection (MCODE) was used to screen sub-networks and hub mRNAs.

### Least Absolute Shrink and Selection Operator-Cox Regression Analysis

In this study, we use the glmnet package in R to integrate age, disease status and gene expression data (from GSE85426 dataset). Then use the lasso-cox method to perform regression analysis. In addition, we also set up 10-fold cross-validation to obtain the optimal model.

### Gene Set Enrichment Analysis

We use the combined gene set of GSE140829 and GSE97760 as the background gene set, and divide patients into high expression group and low expression group according to the expression level of PTGS2 gene. And download GO set, KEGG set and Hallmark gene set from Molecular Signatures Database^7^ to evaluate related pathways and molecular mechanisms. Based on gene expression profile and phenotype grouping, set the minimum gene set to 5 and the maximum gene set to 5000. With one thousand resampling, *p* < 0.05 and FDR < 0.25 were considered statistically significant.

### Cell Culture, Transfection and Treatment

The human neuroblastoma cell line SH-SY5Y was purchased from Beijing Beina Chuanglian Institute of Biotechnology Beijing, (China). SH-SY5Y cell lines were cultured in Duchenne modified Eagle medium (DMEM, Gibco, New York, NY, United States) with 10% FBS, 100 U/ml penicillin/streptomycin (Sigma, St. Louis, MO, United States) in a humidified environment at 37°C with 5% CO_2_. To construct an AD cell model, SHSY5Y cells were treated with 25 μM (Kim et al., 2020) Aβ_25–35_ (Sigma, Shanghai, China) for 24 h. Aβ_25–35_ was dissolved in sterile distilled water at a concentration of 9 mM and then incubated in a bottle at 37°C for 5 days to form a polymer. It is then frozen and stored at 20°C until use. When used, it is diluted to 25 μM. Before Aβ_25–35_ treatment, transfection was performed with Lipofectamine 3000 (Invitrogen, Carlsbad, CA, United States) according to the manufacturer’s instructions. Overexpression plasmid and siRNA, miRNA mimic and miRNA inhibitor are purchased by Ribobio (Guangzhou, China).

### Real-Time qPCR Validation of miR-26a-5p and PTGS2

Peripheral blood samples of 20 non-AD samples and 20 AD patients’ samples (Supplementary Material 1) were collected for qPCR verification to verify the results of bioinformatics analysis. The ethics committee of the Medical Center of the Third Affiliated Hospital of Naval Medical University approved this procedure. Total RNA of peripheral blood was extracted using PAXgene Blood RNA Kit (Qiagen, Düsseldorf, Germany). Total RNA from cells was extracted using AllPrep RNA Universal Kit (Qiagen, Düsseldorf, Germany). Nanodrop 8000 (Thermo, Waltham, MA, United States) was used to detect the RNA concentration. QIAGEN One-Step RT-PCR Kit (Qiagen, Düsseldorf, Germany) was used for reverse transcription and quantitative RT-PCR. GAPDH (for mRNA) and U6 (for miRNA) were used as internal reference controls. The mRNA primers were synthesized by Sangon (China) [PTGS2: forward primer (5′-3′)-AACGCTTTATGCTGAAGCC, Reverse primer (5′-3′)- CCAACTCTGCAGACATTTCC; GAPDH: forward primer (5′-3′)- ATCATCAGCAATGCCTCCT, Reverse primer (5′-3′)- TTCCACGATACCAAAGTTGTC]. The miR-26a-5p primers were purchased by Ribobio (Bulge-Loop hsa-miR-26a-5p Primer Set, China). The relative expression level was calculated using the 2^–ΔΔCt^ method (*n* = 3).

### Western Blot Assay

The processed SH-SY5Y cells were collected, washed with PBS and then extracted with RIPA lysis buffer (Beyotime, Shanghai, China) containing protein inhibitor PMAF. BCA protein concentration determination kit (enhanced) (Beyotime, Shanghai, China) was used to detect protein concentration. The protein sample was boiled at 100°C for 10 min in a 10 × loading buffer (Beyotime, Shanghai, China). SDS-PAGE electrophoresis (Beyotime, Shanghai, China) was performed. After the electrophoresis, the protein was transferred to a polyvinylidene fluoride (PVDF) membrane (Millipore, MA, United States). After the transfer, the PVDF membrane was incubated in 5% skimmed milk for 2 h at room temperature. Then it was placed in the primary antibody (PTGS2: Abcam, Cambridge, United States; β-actin: Beyotime, Shanghai, China) and incubated overnight (4°C). Subsequently, the PVDF membrane was placed in the secondary antibody (Beyotime, Shanghai, China), incubating for 1 h at room temperature. Finally, TMB color developing fluid (Beyotime, Shanghai, China) was used for color development, and then take pictures in the gel imaging system. Subsequently, we analyze the gray value by Image J software.

### Cell Viability Analysis

SH-SY5Y cells were seeded into 96-well plates at a density of 1 × 10^4^ cells/well and incubated for 24 h, then transfected with plasmid and miRNA mimic according to the instructions and incubated for 48 h. After incubation, add 25 μM Aβ_25–35_ to each well for 24 h. Then add 10 μ L CCK-8 solution (Beyotime, Shanghai, China) to each well, and incubate for 3 h. Finally, 450 nm wavelength absorbance was tested on the multifunctional microplate reader. All samples were prepared in triplicate.

### Soft Agar Assay

To analyze the proliferation ability of cells in a 3D environment, we performed a soft agar assay. SH-SY5Y cells were seeded into a 6-well plate (3 × 10^5^ cells per well), and transfected 24 h later. After 48 h of transfection, the medium was removed, and then fresh medium (containing 25 μM Aβ_25–35_) was added and incubated for 24 h. Collect each group of cells, centrifuge and resuspend, count the viable cells, adjust the cell density to 2000 cells/ml with DMEM medium containing 10% FBS. Mix 2000 cells thoroughly with DMEM containing 0.7% agarose, and quickly place them on the solidified layer of DMEM containing 1.2% agarose. Add 500 μl DMEM to each well every 3 days, which contains 10% fetal bovine serum and 25 μM Aβ_25–35_. After culturing for 2 weeks, fix with 4% paraformaldehyde at room temperature, stain with 0.005% crystal violet, take pictures under microscope, count the number of clones larger than 0.05 mm in the field of view and the number of all clones, clone formation rate = number of clones larger than 0.05 mm/all clones Number × 100%.

### Wound Healing Assay

SH-SY5Y cells were seeded into a 6-well plate (3 × 10^5^ cells per well), and transfected 24 h later. After 48 h of transfection, the medium was removed, and then fresh medium (containing 25 μM Aβ_25–35_) was added and incubated for 24 h. Scratch the cell monolayer in a straight line with a 200 μl sterile tip to form a scratch. Phosphate buffered saline (PBS, Beyotime, Shanghai, China) washes suspended cells and cell debris. A fresh medium containing 5% fetal bovine serum was added, and photographed with a microscope after 24 h. Use ImageJ software to evaluate the migration distance. The relative migration rate of each treatment was calculated by the relative migration area of the cells.

### Dual-Luciferase Assay

The luciferase reporter plasmids pmiR-RB-Report™-PTGS2-wt and PMIR- RB- Report™-PTGS2-mut were purchased from Ribobio (Guangzhou, China), and then co-transfected into SH-SY5Y cells with miR-26a-5p mimic, respectively. Dual-luciferase reporting kit (Promega, Madison, WI, United States) was used for detection after 48 h transfection.

### Network Pharmacology Analysis

First, we obtain AD-related Chinese herbal medicines and Chinese herbal medicines targeting PTGS2 from HERB^8^ and ETCM database^9^. Then obtain candidate Chinese herbal medicines through Venn diagram. Subsequently, the HERB database was used to obtain the active ingredients of key Chinese herbal medicines and the active ingredients targeting PTGS2. Then use the TCMSP database^10^ to screen the main active ingredients of key Chinese herbal medicines according to the standard OB ≥ 30%, DL ≥ 0.18 (Jing et al., 2019). Finally, the candidate active ingredients of Chinese herbal medicine were obtained through Venn diagram again.

### Drug Preparation and Treatment

Baicalein (purity 98%) was purchased from Sigma (St. Louis, MO, United States). Use dimethyl sulfoxide (DMSO) to dissolve baicalein to 2.5 mg/ml, and then dilute with medium. The final concentration of DMSO in the baicalein treatment group was less than 0.05%. Before Aβ_25–35_ (25 μM) stimulates SH-SY5Y cells, SH-SY5Y cells were pretreated with 7.5, 12.5, 25, 50, and 100 μg/ml respectively. For SY5Y cells for 2 h, the negative control was a medium containing 0.05% DMSO.

### Statistical Analysis

GraphPad Prism 8 was used for statistical analysis. The data are expressed as mean ± standard deviation (SD). The difference between the two groups was tested by Student’s *t*-test, and the one-way analysis of variance was used to analyze the data of more than two groups. *p* < 0.05 was considered statistically significant.

## Results

### Identification of Differentially Expressed miRNAs and mRNAs in Alzheimer’s Disease

**FIGURE 1:**
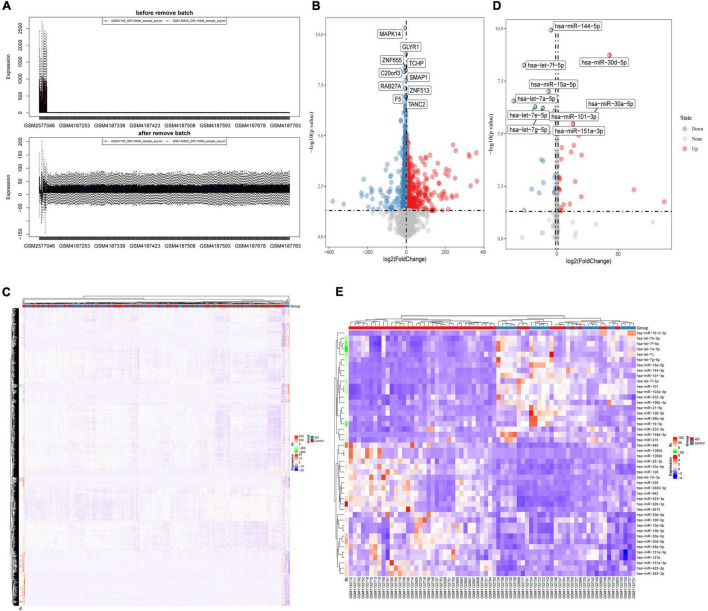
**(A)** The SVA package removes the batch effect between GSE140829 and GSE97760. **(B)** Volcano plot and **(C)** heatmap for the differentially expressed mRNAs in the merged dataset (GSE140829 and GSE97760). **(D)** Volcano plot and **(E)** heatmap for the differentially expressed miRNAs in GSE46579. Red dots represent up regulation, blue dots represent down regulation, and gray dots represent non-differentially expressed RNA.

After merging the 2-mRNA expression profile (GSE140829 and GSE97760), we use the SVA package to remove batch effects between datasets (Figure 1A). According to the screening criteria for differentially expressed mRNAs, we obtained 2754 differentially expressed mRNAs (1740 down-regulated and 1014 up-regulated) from the processed expression profile (Table 2). Similarly, based on the same criteria, 46 differentially expressed miRNAs (21 down-regulated and 25 up-regulated) were screened from the expression profile of GSE46579 (Table 3). Heat map and volcano map were used to display differentially expressed mRNAs (Figures 1B,C) and differentially expressed miRNAs (Figures 1D,E).

**TABLE 2:** Top10 (up- and down-regulated) of differentially expressed mRNAs in Alzheimer’s disease expression profile.

Symbol	logFC	AveExpr	*t*	*p*-Value
**Down regulation**				
S100A8	–379.35525	2661.710738	–2.38487964	0.01747973
RPL41	–323.34655	2526.996787	–2.25484895	0.0246017
RPL36A	–231.79430	923.623438	–2.72251730	0.00671923
RPS29	–231.29417	1855.841695	–2.59834178	0.00966134
LCE1D	–220.17993	937.7317378	–3.20582535	0.00143839
RPS27	–204.75933	1621.477865	–2.6152558	0.00920233
RPL35A	–195.92404	1856.677681	–2.13995778	0.0328718
RPS7	–182.71717	737.7545075	–2.90099666	0.00389406
RPL34	–182.21577	578.4318178	–2.73218254	0.00652828
RPL26	–175.81143	597.5430597	–2.81186837	0.00513123
**Up regulation**				
SORBS3	237.93649	743.5955336	2.54796438	0.01115217
GUK1	240.66325	895.0004549	4.22763152	2.84E-05
FTH1	250.53080	2034.69856	3.24611638	0.00125336
BLVRB	254.47774	1067.233439	4.07245999	5.46E-05
IFITM2	258.69525	2384.606542	2.61461587	0.00921934
HBQ1	321.65242	858.7720207	3.96278955	8.56E-05
TESC	324.84050	1312.397636	3.90843923	0.00010653
TYROBP	331.14476	3256.789916	2.97956045	0.00303593
SLC25A39	360.45545	1214.226044	4.01435967	6.93E-05
PTMS	363.48563	1331.152503	3.13891648	0.00180229

**TABLE 3:** Top 10 (up- and down-regulated) of differentially expressed miRNAs in Alzheimer’s disease expression profile.

Symbol	logFC	AveExpr	*t*	*P*-value
**Down regulation**				
hsa-let-7a-5p	–35.491	82.403010	–5.70087	2.77E-07
hsa-let-7f-5p	–27.116	70.260326	–6.66472	5.53E-09
hsa-miR-16-5p	–26.797	78.130188	–2.05364	0.04383331
hsa-let-7c	–20.487	56.909060	–2.95529	0.00428107
hsa-let-7e-5p	–17.824	43.261480	–5.54057	5.22E-07
hsa-let-7i-5p	–14.485	34.235053	–2.80225	0.00659440
hsa-miR-103a-3p	–12.598	29.050312	–3.97604	0.00017138
hsa-let-7g-5p	–11.700	16.084993	–5.50547	5.99E-07
hsa-miR-107	–11.157	25.335093	–3.92486	0.00020394
hsa-miR-215	–11.028	21.214721	–3.19202	0.00213464
**Up regulation**				
hsa-miR-151a-3p	13.039	23.205877	5.059287	3.37E-06
hsa-miR-186-5p	13.794	26.996634	4.423297	3.57E-05
hsa-miR-26a-5p	14.948	40.893653	3.235016	0.00187465
hsa-miR-425-5p	17.859	35.817214	4.718769	1.21E-05
hsa-miR-423-3p	19.945	55.096995	2.651547	0.00994768
hsa-miR-363-3p	20.187	48.662844	4.117417	0.00010536
hsa-miR-30a-5p	27.306	70.401440	5.214316	1.86E-06
hsa-miR-30d-5p	42.808	79.927921	6.931773	1.83E-09
hsa-miR-484	62.902	152.71103	2.927451	0.00463608
hsa-miR-92b-3p	87.638	263.71002	2.436479	0.01743737

### Potential miRNAs Related to Alzheimer’s Disease Occurrence Identified via Weighted Gene Co-expression Network Analysis

**FIGURE 2:**
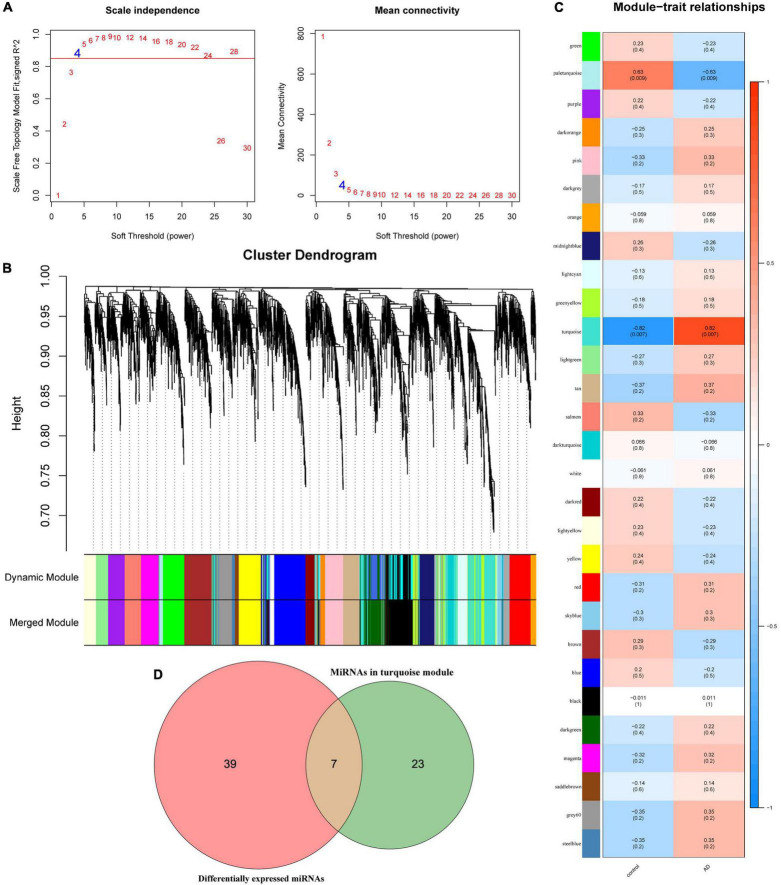
Weighted gene co-expression network analysis analysis results in GSE157239 dataset. **(A)** Analysis of the scale-free index for various soft-threshold powers (β) and the mean connectivity for various soft-threshold powers. **(B)** Dendrogram of all miRNAs clustered in GSE157239. **(C)** Heatmap of the correlation between the module eigengenes and clinical traits of Alzheimer’s disease. **(D)** Venn diagram shows the overlapping miRNAs in the turquoise module and differentially expressed miRNAs.

Weighted gene co-expression network analysis analysis is performed on the samples of the GSE157239 dataset. The results show that the miRNA co-expression network conforms to the scale-free feature network. The best soft threshold β = 4 (*R*^2^ = 0.85, Figure 2A). The average linkage hierarchical clustering method divides all miRNAs into 29 modules (Figure 2B). According to the module eigengene (ME) value of the modules, the correlation between these modules and the phenotype is calculated. The results show that the turquoise module has the greatest correlation with the sample type (correlation coefficient is 0.82, *p* = 0.007) (Figure 2C), indicating that the miRNAs in the turquoise module (*n* = 30) obtained by WGCNA may be related to the pathogenesis of AD. Subsequently, we compared the differentially expressed miRNAs and miRNAs in the turquoise module through the Venn diagram, and obtained 7 overlapping miRNAs (hsa-miR-30a-5p, hsa-miR-26a-5p, hsa-miR-151a-5p, hsa-miR-101-3p, hsa-let-7e-5p, hsa-miR-15a-5p and hsa-let-7i-5p) (Figure 2D), which means that these 7 miRNAs may be more related to the pathogenesis of AD.

### Expression Validation and Diagnostic Analysis of 7 Overlapping miRNAs

**FIGURE 3:**
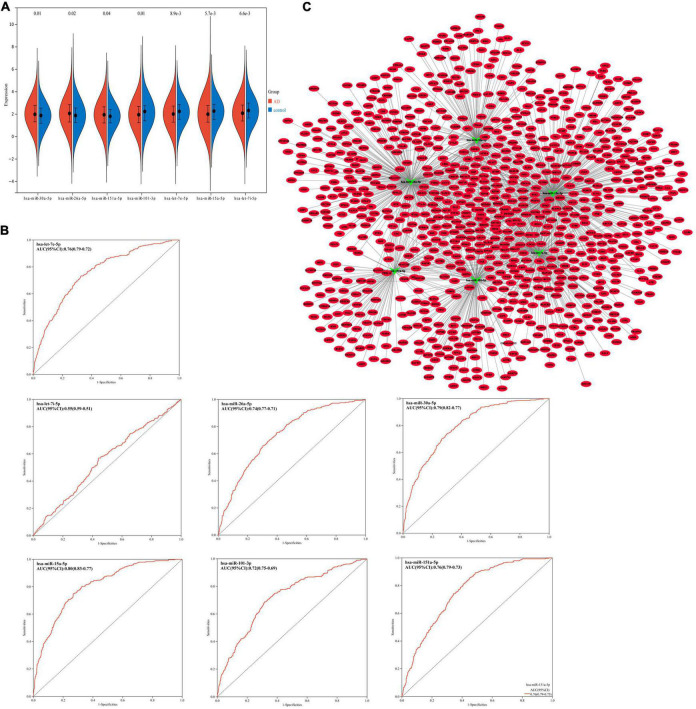
**(A)** Expression trends of hsa-miR-30a-5p, hsa-miR-26a-5p, hsa-miR-151a-5p, hsa-miR-101-3p, hsa-let-7e-5p, hsa-miR-15a-5p and hsa-let-7i-5p in GSE120584. **(B)** Receiver operator characteristic curve (ROC) analysis of hsa-miR-30a-5p, hsa-miR-26a-5p, hsa-miR-151a-5p, hsa-miR-101-3p, hsa-let-7e-5p, hsa-miR-15a-5p, and hsa-let-7i-5p base on GSE120584 dataset. AUC, Area Under the Curve. **(C)** miRNA–mRNA regulatory network. The green triangle represents miRNA and the red oval represents miRNA.

The GSE120584 data set was used for expression level verification and ROC analysis of overlapping miRNAs. Compared with the normal control group, the expression of hsa-miR-30a-5p, hsa-miR-26a-5p, and hsa-miR-151a-5p were up-regulated (*p* < 0.05), and the other 4 miRNAs were down-regulated (*p* < 0.05) (Figure 3A). This result is consistent with that of differential expression screening. In ROC analysis, except for hsa-let-7i-5p (AUC = 0.55), the area under the curve (AUCs) of the other six miRNAs were > 0.7 (Figure 3B). This indicates that these six miRNAs can be potential diagnostic markers for AD.

### Construction of miRNA–mRNA Regulatory Network

Using the miRWalk database and Starbase database to predict the six key miRNAs (miR-30a-5p, miR-26a-5p, miR-151a-5p, miR-101-3p, let-7e-5p, miR-15a-5p) target gene. Combined with target genes and differentially expressed mRNAs, a total of 863 mRNAs were obtained (Supplementary Table 1). According to the regulatory relationship between miRNA and miRNA, a miRNA-mRNA interaction network consisting of 869 nodes and 1360 edges was constructed (Figure 3C).

### Kyoto Encyclopedia of Genes and Genomes and Gene Ontology Enrichment Analysis for mRNAs in miRNA–mRNA Regulatory Network

**FIGURE 4:**
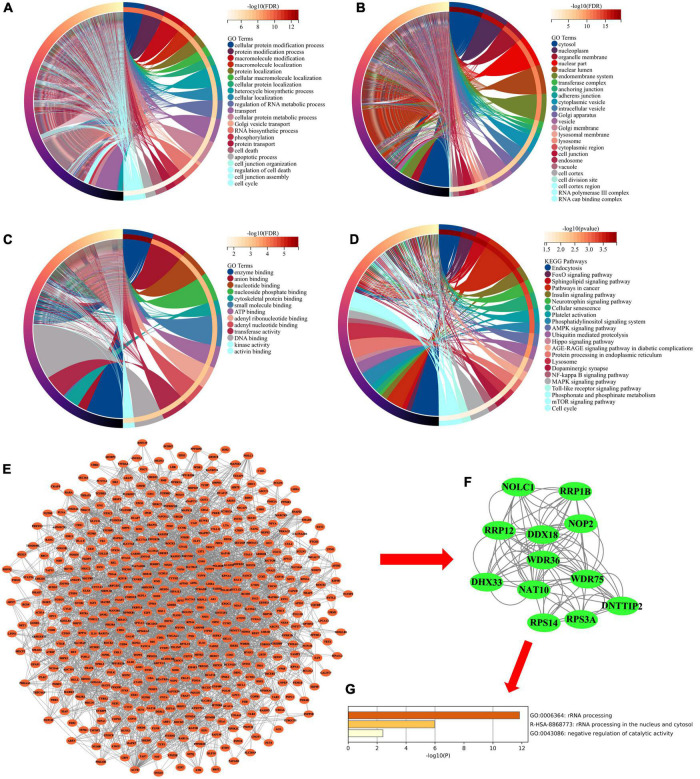
**(A)** Chord diagram showing enriched GO-BP clusters for the mRNAs in miRNA-mRNA regulatory network. BP, biological processes. **(B)** Chord diagram showing enriched GO-CC clusters for the mRNAs in miRNA-mRNA regulatory network. CC, cell composition. **(C)** Chord diagram showing enriched GO-MF clusters for the mRNAs in miRNA-mRNA regulatory network. MF, molecular functions. **(D)** Chord diagram showing enriched KEGG clusters for the mRNAs in miRNA-mRNA regulatory network. GO, Gene Ontology; KEGG, Kyoto Encyclopedia of Genes and Genomes. **(E)** PPI network analysis for the mRNAs in miRNA-mRNA regulatory network. PPI, protein–protein interaction. **(F)** The hub subnetwork from PPI network by Molecular Complex Detection (MCODE) plug-in. **(G)** Functional enrichment analysis of mRNAs in hub subnetwork.

The 863 common mRNAs in the miRNA–mRNA regulatory network were analyzed by DAVID enrichment (*p* < 0.05). GO enrichment analysis shows that in biological processes, these mRNAs are mainly involved in protein modification and localization, protein transportation and metabolism, cell necrosis, cell cycle and apoptosis (Figure 4A). From the perspective of cell composition, these mRNAs are mainly distributed in the cytoplasm, nucleoplasm, inner membrane system, vesicles, lysosomes and cell junctions (Figure 4B). In terms of molecular functions, these mRNAs are mainly involved in nucleotide binding, enzyme binding, DNA binding, ATP binding and kinase activation (Figure 4C). In addition, KEGG enrichment analysis found that these mRNAs are involved in endocytosis, Neurotrophin signaling pathway, cell senescence, platelet activation, phosphatidylinositol signaling system, AMPK signaling pathway, lysosome and NF-kappa B signaling pathway (Figure 4D).

### Protein–Protein Interaction Network Construction and Hub mRNAs Screening

**FIGURE 5:**
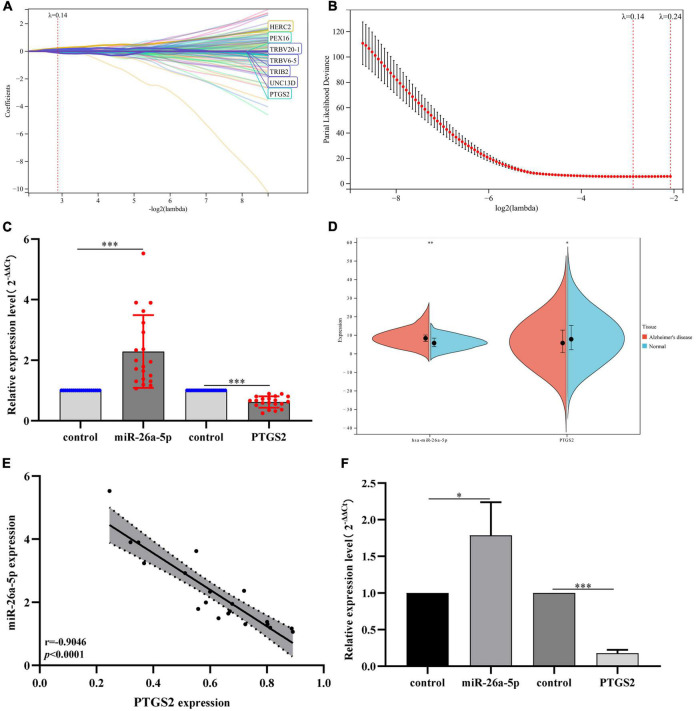
**(A)** LASSO coefficient profiles of 7 AD characteristic genes in the GSE85426 dataset. **(B)** Plot of partial likelihood deviance. **(C)** The expression levels of miR-26a-5p and PTGS2 in peripheral blood of AD patients (20 AD patients and 20 control samples). The expression level of GAPDH was used as an internal reference for PTGS2 and U6 for miR-26a-5p. The data are shown as the mean ± standard deviation. **(D)** The expression levels of miR-26a-5p and PTGS2 in the merged dataset (GSE140829 and GSE97760). **(E)** Correlation analysis of the expression of miR-26a-5p and PTGS2 in peripheral blood of AD patients (20 AD patients and 20 control samples). **(F)** The expression levels of miR-26a-5p and PTGS2 in AD model cells (Aβ_25–35_-treated SH-SY5Y cells). The expression level of GAPDH was used as an internal reference for PTGS2 and U6 for miR-26a-5p. The data are shown as the mean ± standard deviation. AD, Alzheimer’s disease. ^***^p < 0.001; ^**^p < 0.01; *p < 0.05.

Subsequently, we performed PPI network analysis on 863 common mRNAs to explore the interaction of these mRNAs and the hub gene. Use STRING to calculate intersecting mRNAs and build a PPI network. Then use Cytoscape software to visualize the interaction pairs with a confidence score > 0.7 (Figure 4E). As shown in Figure 4E, the network has 491 nodes and 2300 edges. Subsequently, a hub sub-network (Figure 4F) is obtained through the plug-in MCODE in Cytoscape, which contains 12 nodes and 92 edges. The results of functional enrichment analysis showed that mRNAs in the hub network are mainly involved in metabolic processes, biological process regulation, and rRNA processing (Figure 4G). Furthermore, we downloaded the GSE85426 dataset from the GEO database. Then through LASSO regression for feature screening, 7 AD feature genes were obtained (λ = 0.136, Figures 5A,B). Subsequently, the 12 mRNAs and 7 AD characteristic genes in the hub sub-network were compared, and 2 crossover genes (TRIB2 and PTGS2) were obtained (Supplementary Table 2). These results indicate that the 2 genes (TRIB2 and PTGS2) may play a more important role in the pathogenesis of AD. In the hub sub-network, the degree of connection of PTGS2 (degree = 22) is greater than that of TRIB2 (degree = 16), so PTGS2 is selected as the candidate gene for subsequent analysis. Similarly, in the miRNA–mRNA network, among the miRNAs that interact with PTGS2, hsa-miR-26a-5p (degree = 269) has the highest degree of connectivity, so miR-26a-5p/PTGS2 is selected as the research role of this project.

### RT-PCR Validation of miR-26a-5p/PTGS2 and Gene Set Enrichment Analysis Enrichment Analysis of PTGS2

The clinical peripheral blood sample information in this study is shown in Supplementary Material 1. The expression levels of miR-26a-5p and PTGS2 in blood samples of AD patients were analyzed by RT-qPCR. qPCR results showed that compared with the normal control group, the expression of miR-26a-5p in the blood of AD patients was up-regulated, while the expression of PTGS2 was down-regulated (Figure 5C). The relative expression trends of mRNA and miRNA are consistent with bioinformatics analysis (Figure 5D), and there is a significant difference in the expression level (*p* < 0.05). The correlation analysis results showed that the expression levels of miR-26a-5p and PTGS2 showed a significant negative correlation (*p* < 0.05) (Figure 5E). Moreover, we also used qRT-PCR to detect the expression levels of miR-26a-5p and PTGS2 in AD model cells (Aβ_25–35_-treated SH-SY5Y cells). Compared with the untreated group, the expression of miR-26a-5p in Aβ_25–35_-treated SH-SY5Y cells was significantly up-regulated (*p* < 0.05), and the expression of PTGS2 was significantly decreased (*p* < 0.05) (Figure 5F). These results are consistent with the trend of expression in clinical peripheral blood.

**FIGURE 6:**
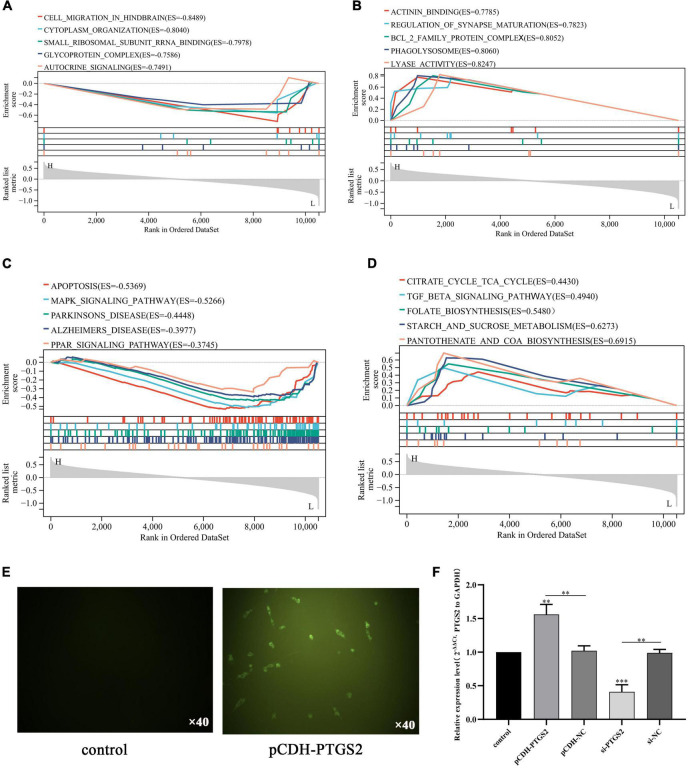
Gene set enrichment analysis revealed five positively correlated GO term with lower expression PTGS2 **(A)**, five negatively correlated GO term with lower expression PTGS2 **(B)**, five positively correlated KEGG pathway with lower expression PTGS2 **(C)**, and five negatively correlated KEGG pathway with lower expression PTGS2 **(D)**. **(E)** The transfection efficiency of pCDH-PTGS2 was detected by fluorescence microscope. Scales: 10 × 40. **(F)** The transfection efficiency of pCDH-PTGS2 and si-PTGS2 was detected by qPCR. The expression level of GAPDH was used as an internal reference for PTGS2. The data are shown as the mean ± standard deviation. The horizontal line indicates the comparison between groups at both ends, and the non-horizontal line indicates the comparison with the control group. ^***^p < 0.001; ^**^p < 0.01.

Furthermore, we used the combined gene set of GSE140829 and GSE97760 as the background gene set, and used GO term and KEGG pathway in Gene Set Enrichment Analysis (GSEA) to analyze the potential biological functions of PTGS2. We select the most enriched signaling pathway based on its enrichment score (ES). As shown in Figure 6A, GO annotation reveals five categories that are positively correlated with low PTGS2 expression: autocrine signaling, glycoprotein complex, small ribosomal subunit rRNA binding, cytoplasm organization and cell migration in hindbrain. GO analysis also found five types of negative correlations: actinin binding, regulation of synaptic maturation, Bcl2 family protein complex, phagolysosome and lyase activity (Figure 6B). KEGG pathway analysis showed that the five pathways with the strongest positive correlation with PTGS2 expression: apoptosis, MAPK signaling pathway, Parkinson’s disease, AD and PPAR signaling pathway (Figure 6C). It also found five types of negative correlations for KEGG pathway analysis correlation with PTGS2 expression: coenzyme synthesis, glycometabolism, folic acid biosynthesis, Citric acid cycle TCA cycle and TGF beta signaling pathway (Figure 6D).

### The Effect of PTGS2 Expression on Alzheimer’s Disease Model Cells Proliferation and Migration

**FIGURE 7:**
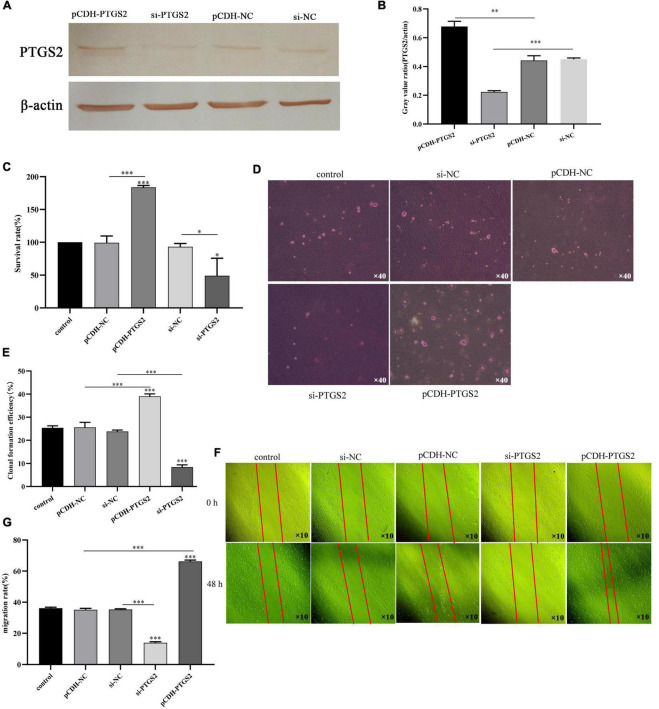
**(A,B)** The transfection efficiency of pCDH-PTGS2 and si-PTGS2 was detected by western blot. **(C)** The effects of PTGS2 knockdown or overexpression on the vitality of AD model cells (Aβ_25–35_-treated SH-SY5Y cells) measured using the CCK-8 assay. **(D,E)** The effects of PTGS2 knockdown or overexpression on the proliferation of AD model cells (Aβ_25–35_-treated SH-SY5Y cells) measured using the soft agar assay. Scales: 10 × 40. **(F,G)** The effects of PTGS2 knockdown or overexpression on the migration of AD model cells (Aβ_25–35_-treated SH-SY5Y cells) measured using the wound healing assay. Scales: 10 × 10. The data are shown as the mean ± standard deviation. The horizontal line indicates the comparison between groups at both ends, and the non-horizontal line indicates the comparison with the control group. AD: Alzheimer’s disease. ^***^p < 0.001; ^**^p < 0.01; *p < 0.01.

To determine the functional significance of PTGS2 in the damage of AD model cells (Aβ_25–35_-treated SH-SY5Y cells), a gain-of-function experiment was performed in AD model cells. Firstly, PTGS2 overexpressed plasmid was transfected into AD model cells, and fluorescence was observed under fluorescence microscope (Figure 6E). At the same time, qPCR results showed that the expression of PTGS2 in the overexpressed group was significantly up-regulated, and the expression of PTGS2 in the silent group was significantly inhibited (Figure 6F). Western blot results show the same results (Figures 7A,B). These results indicate that the plasmid transfection was successful. CCK-8 assay results showed that PTGS2 overexpression can significantly increase the proliferation activity of AD model cells. Silencing the expression of PTGS2 showed the opposite result (Figure 7C). The soft agar assay also showed that overexpression of PTGS2 promoted the clone formation, while inhibiting the expression of PTGS2 weakened the ability of clone formation (Figures 7D,E). The wound healing assay showed the same trend. pCDH-PTGS2 can significantly increase the migration rate of AD model cells (Figures 7F,G).

### PTGS2 Is Involved in the Regulatory Effects of miR-26a-5p on Alzheimer’s Disease Model Cells

**FIGURE 8:**
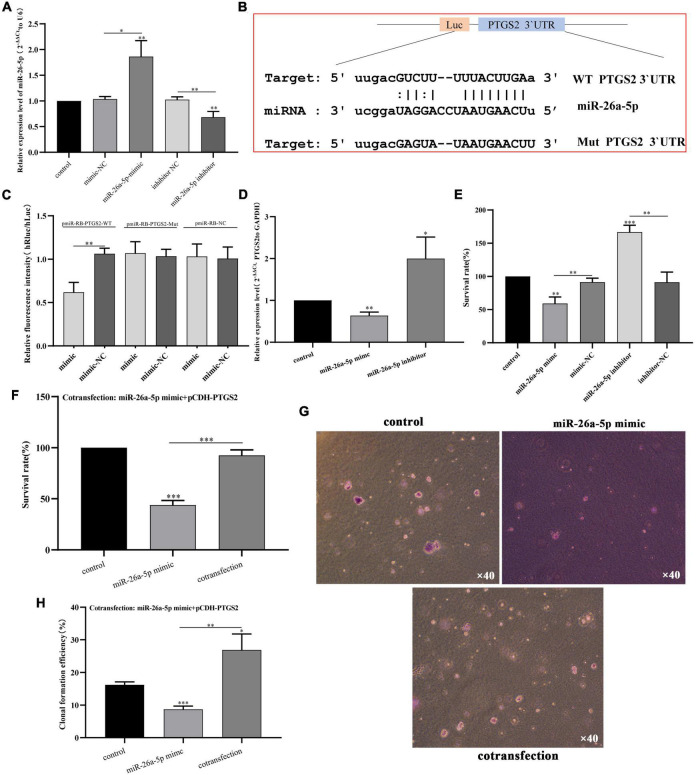
**(A)** The transfection efficiency of miR-26a-5p (miR-26a-5p mimic and miR-26a-5p inhibitor) was detected by qPCR. The expression level of U6 was used as an internal reference for miR-26a-5p. The data are shown as the mean ± standard deviation. **(B)** Binding site sequences of miR-26a-5p and PTGS2 3 ‘UTR. **(C)** Luciferase activity of WT or Mut PTGS2 after co-transfection of miR-26a-5p mimic and WT or Mut PTGS2 dual-fluorescent vector into SH-SY5Y cells. **(D)** The effects of miR-26a-5p mimic and mir-26a-5p inhibitor on the expression of PTGS in AD model cells (Aβ_25–35_-treated SH-SY5Y cells) were detected by qPCR. **(E)** The effects of miR-26a-5p mimic and miR-26a-5p inhibitor on the vitality of AD model cells (Aβ_25–35_-treated SH-SY5Y cells) measured using the CCK-8 assay. **(F)** The effects of cotransfection (pCDH-PTGS2 and miR-26a-5p mimic) on the vitality of AD model cells (Aβ_25–35_-treated SH-SY5Y cells) measured using the CCK-8 assay. **(G,H)** The effects of cotransfection (pCDH-PTGS2 and miR-26a-5p mimic) on the proliferation of AD model cells (Aβ_25–35_-treated SH-SY5Y cells) measured using the soft agar assay. Scales: 10 × 40. The data are shown as the mean ± standard deviation. The horizontal line indicates the comparison between groups at both ends, and the non-horizontal line indicates the comparison with the control group. AD, Alzheimer’s disease. ^***^p < 0.001; ^**^p < 0.01; *p < 0.01.

First, we identified the transfection efficiency of miR-26a-5p by qPCR. The relative expression of miR-26a-5p increased significantly after miR-26a-5p mimic transfection (*p* < 0.05), and decreased after miR-26a-5p inhibitor transfection (*p* < 0.05) (Figure 8A). PTGS2 and miR-26a-5p was evaluated by bioinformatics analysis and dual-luciferase experimental analysis. As shown in Figure 8B, Target scan predicted the binding sites of PTGS2 and miR-26a-5p online, suggesting that PTGS2 may be the target of miR-26a-5p. To verify this prediction, PTGS2-WT and PTGS2-Mut plasmids were constructed and co-transfected with miR-26a-5p mimic into AD model cells. The results of dual-luciferase analysis showed that the luciferase activity of the PTGS2-WT group was significantly reduced, while the luciferase activity of the PTGS2-Mut group did not change (Figure 8C). Subsequently, qPCR results showed that overexpression of miR-26a-5p can significantly reduce the expression level of PTGS2 in AD model cells, while downregulation of miR-26a-5p can significantly increase the expression level of PTGS2 (Figure 8D).

Furthermore, in order to explore the role of miR-26a-5p in AD model cells, miR-26a-5p mimic or miR-26a-5p inhibitor was transfected into AD model cells. After 48 h of treatment, the up-regulation of miR-26a-5p weakened the proliferative activity of AD model cells, while silencing had the opposite effect (Figure 8E). CCK-8 experiments showed that up-regulation of PTGS2 can improve miR-26a-5p-mediated cell viability in AD model cells (Figure 8F). The soft agar assay also shows the same trend. The expression of PTGS2 can interfere with the number of clones regulated by miR-26a-5p (Figures 8G,H).

### PTGS2-Related Chinese Medicine Active Component Analysis

**FIGURE 9:**
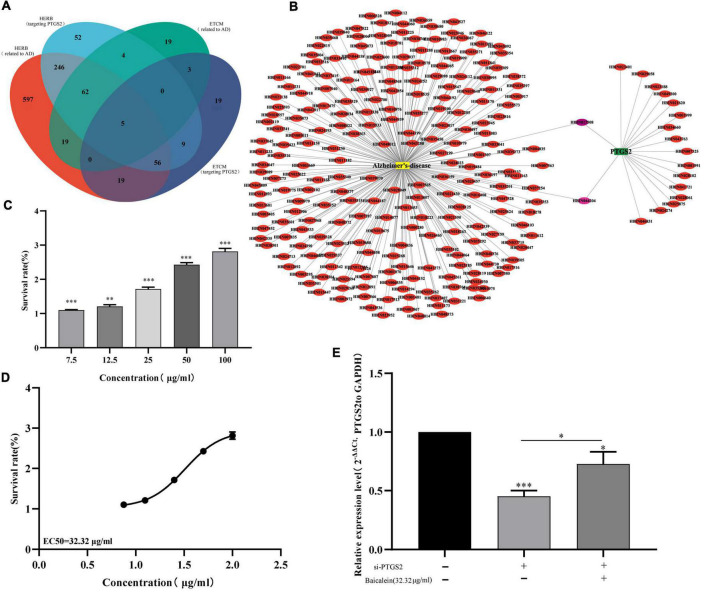
**(A)** Venn diagram shows the overlap between AD related Chinese herbs and PTGS related Chinese herbs. **(B)** Network of AD related components in HUANGQIN and PTGS2 targeted components in HUANGQIN. **(C)** The effects of different concentrations baicalein on the vitality of AD model cells (Aβ_25–35_-treated SH-SY5Y cells) measured using the CCK-8 assay. **(D)** EC_50_ fitting curve of baicalein in AD model cells (Aβ_25–35_-treated SH-SY5Y cells). **(E)** The effect of baicalein on the expression of PTGS2 by qPCR in AD model cells (Aβ_25–35_-treated SH-SY5Y cells). AD, Alzheimer’s disease. The expression level of GAPDH was used as an internal reference for PTGS2. The data are shown as the mean ± standard deviation. The horizontal line indicates the comparison between groups at both ends, and the non-horizontal line indicates the comparison with the control group. ^***^p < 0.001; *p < 0.05.

Based on the traditional Chinese medicine pharmacology database, we analyzed the Chinese herbal medicines targeting PTGS2, and further analyzed the main active ingredients targeting PTGS2 in the candidate Chinese herbal medicines. As show in Supplementary Material 2, 1004 and 111 Chinese herbal medicines related to AD were obtained from HERB and ETCM databases, respectively. Also, 434 and 112 Chinese herbal medicines targeting PTGS2 were obtained from HERB and ETCM databases, respectively. Five overlapping Chinese herbal medicines (CANG ER ZI, CE BAI YE, GUANG HUO XIANG, HUANG QIN, and WU ZHU YU) were obtained by Venn diagram comparison (Figure 9A). We chose HUANG QIN, which is commonly used in traditional Chinese medicine, for further analysis. The HERB database shows that HUANG QIN contains 233 kinds of ingredients (Supplementary Material 3). Similarly, 18 Chinese herbal ingredients related to PTGS2 were obtained from HERB Database (Supplementary Material 4). Then, 2 common ingredients of traditional Chinese medicine (HBIN044204: s-methyl mercapto-l-cysteine and HBIN017508: baicalein) were obtained by comparison (Figure 9B). Subsequently, 38 main active ingredients contained in HUANG QIN were screened through TCMSP database (Supplementary Material 5). By comparison, we found that baicalein in the 2 candidate ingredients of traditional Chinese medicine (s-methyl mercapto-l-cysteine and baicalein) was one of the 38 main active ingredients in the HUANG QIN. These results suggest that baicalein may affect the progress of AD by regulating PTGS2.

Furthermore, different concentrations of baicalein (0, 7.5, 12.5, 25, 50, and 100 μg/ml) were used to treat AD model cells, CCK-8 assay showed that baicalein can significantly increase the activity of AD model cells (Figure 9C). The results of fitting curve analysis showed that the EC_50_(median effective concentration) of baicalein in model cells was 32.32 μg/ml (Figure 9D). RT-qPCR results further showed that baicalein can increase the expression level of PTGS2 to a certain extent (Figure 9E). These results indicate that baicalein can not only affect the proliferation activity of AD model cells, but also affect the expression of PTGS2, which indicates that baicalein may be able to affect the occurrence or deterioration of AD by regulating the expression of PTGS2.

## Discussion

Alzheimer’s disease is the most common form of dementia in the elderly, which can cause orientation disorders, memory loss and visual spatial ability in the elderly (Alzheimer’s Association, 2022). Despite great efforts to study the causes of AD, AD is still the fifth leading cause of death in the world (Wang L. et al., 2020). According to reports, patients with AD have neuropathology in the brain before they develop symptoms (Miao et al., 2020). Therefore, it is very important to develop new and practical biomarkers for early diagnosis of AD. At present, various RNAs have become the focus of research on human disease mechanisms, including mRNA, miRNA, and lncRNA (Wolin and Maquat, 2019; Barbieri and Kouzarides, 2020). In this study, mRNAs and miRNAs that may affect the progression of AD were analyzed by a series of bioinformatics techniques and experimental methods, and investigated the regulatory role of key pair miR-26A-5p/PTGS2 in AD model cells and possible effective targeting of Chinese herbal active ingredients.

miRNA is a small endogenous non-coding RNA that negatively regulates gene expression at the post-transcriptional level by binding to the 3′UTR of its target mRNA and affects various biological processes such as cell proliferation, differentiation, apoptosis and cycle (Ali Syeda et al., 2020; Yoshida et al., 2021). miRNA with abnormal expression patterns has been identified as a key molecule in the pathogenesis of various human diseases, including AD and cancer (Ali Syeda et al., 2020; Serpente et al., 2020; Yoshida et al., 2021). In the development and progress of AD, some functional miRNAs have also been discovered in recent decades. Research by Ji et al. (2019) showed that miR22-3p is down-regulated in AD and can inhibit Aβ deposition by targeting MAPK14. Shi et al. (2020) found that in AD patients, the abnormal increase of miR-34c can target synaptotagmin 1 through the ROS-JNK-p53 pathway to mediate synaptic defects. Wang et al. (2018) also showed that miRNA-124 targeting PTPN1 mediates synaptic and memory defects in AD through a new signaling pathway. The above studies have shown that abnormally expressed miRNAs may provide new ideas for the treatment of AD by regulating key genes in the pathogenesis of AD. In our study, RNA expression profiles and miRNA expression profiles of AD patients were downloaded from GEO database and analyzed by differential expression analysis, WGCNA, miRNA-mRNA regulatory Network, PPI Network, ROC and LASSO Regression and other bioinformatics methods, we screened the key miRNA-mRNA in the progress of AD: miR-26a-5p/PTGS2. RT-qPCR detection showed that the expression of miR-26a-5p was up-regulated and the expression of PTGS2 was down-regulated in the blood samples of AD patients. The relative expression trends of mRNA and miRNA are consistent with bioinformatics analysis. Moreover, miR-26a-5p was significantly negatively correlated with the expression level of PTGS2. In addition, we also found that the expression trend of miR-26a-5p and PTGS2 in AD model cells (SH-SY5Y cells treated with Aβ_25–35_) was consistent with the clinical peripheral blood detection results. The above results indicate that miR-26a-5p and PTGS2 are abnormally expressed RNAs in the occurrence and development of AD.

Prostaglandin endoperoxidase synthase 2 (PTGS2) is one of the key enzymes mediating the new synthesis of prostaglandin, which has been proved to have the functions of inducing apoptosis, inhibiting proliferation and inhibiting metastasis (Nagaraju and El-Rayes, 2019; Spence et al., 2019; Tan et al., 2019). Frejborg et al. (2020) reported that PTGS2 can promote the development and progression of head and neck cancer through various mechanisms such as inducing cell proliferation, inhibiting cell apoptosis, and inhibiting host immune response. In the human brain, PTGS2 is mainly expressed in the cerebral cortex, hippocampus, hypothalamus, thalamus, cerebellum and other key parts. It is often observed to be released by astrocytes, microglia, brain endothelial cells and peripheral immune cells, which affect the function of the brain and nerve excitability (Rawat et al., 2020; Prabhakaran et al., 2021). Therefore, PTGS2 may have important significance in behavior and cognitive function, and may be a key target of brain diseases. In this project, the gain-of-loss function experiment also confirmed that the overexpression of PTGS2 can significantly improve the proliferation activity and migration rate of AD model cells. Bioinformatics and dual-luciferase reporter gene detection showed that PTGS2 is the direct target gene of miR-26a-5p. Overexpression of miR-26a-5p can significantly reduce PTGS2 mRNA and protein levels in AD model cells, while downregulation of miR-26a-5p can significantly increase PTGS2 protein levels. According to reports, miR-26a-5p is a classic molecule of the miRNA family and has been identified as a tumor suppressor, which has an important anti-proliferation effect in certain malignant tumors (Wang Z. et al., 2020). For example, in hepatocellular carcinoma, the down-regulation of miR-26a-5p promotes tumor metastasis and is associated with poor prognosis (Chang et al., 2017). In AD, miR-26a-5p in peripheral blood is considered to be a diagnostic marker (Targa et al., 2020), but the specific function it performs has not yet been studied in detail. Liu et al. (2020) found in mice that miR-26a-5p mimic inhibits Tau phosphorylation and β accumulation in AD mice by targeting DYRK1A. However, the function of miR-26a-5p in AD patients has not yet been reported. In this study, we found that miR-26a-5p up-regulates the proliferation activity of AD model cells, while miR-26a-5p inhibitors do the opposite. The overexpression of PTGS2 can not only rescue the change of PTGS2 protein, but also improve the cell viability of AD model cells mediated by miR-26a-5p. Collectively, these results indicate that miR-26a-5p directly targets PTGS2 and plays a regulatory role in AD.

Network pharmacology is based on the interaction of component-target-disease association networks. Screening effective drugs corresponding to disease targets through this complex network can accurately identify gene-targeted Chinese medicine components (Wu et al., 2019). Based on Venn diagram analysis, five Chinese herbal medicines (CANG ER ZI, CE BAI YE, GUANG HUO XIANG, HUANG QIN, and WU ZHU YU) were found to be common between targeted PTGS2 and AD. Further analysis showed that baicalein was the main component related to PTGS2. CCK-8 showed that baicalein could significantly improve the proliferation of AD model cells. RT-qPCR results further showed that baicalein could increase the expression level of PTGS2. These results suggest that baicalein may affect the progression of AD by regulating PTGS2. Ji et al. (2020) found that baicalein-mediated regulation of NF-κB/MAPK pathway has a protective effect on amyloid-induced toxicity of nerves. Dhakal et al. (2021) also found that baicalein reduced intracellular amyloid-induced oxidative damage in a yeast model of AD. In a word, baicalein can effectively affect the progression of AD, but the specific regulatory mechanism needs to be further studied.

## Conclusion

In our study, we conducted bioinformatics analysis based on AD RNA expression profile data in GEO, through differential expression analysis, WGCNA, miRNA–mRNA regulatory Network, PPI Network, ROC and LASSO Regression, Correlation analysis, qPCR and dual luciferase analysis methods, we screened the key miRNA-mRNA in the progress of AD: miR-26a-5p/PTGS2. Cell experiments confirmed that PTGS2 can protect AD model cells (Aβ_25–35_-treated SH-SY5Y cells) from damage by inhibiting proliferation and migration. In addition, overexpression of PTGS2 can not only inhibit the expression of miR-26a-5p, but also reverse the damage of miR-26a-5p to AD model cells. Furthermore, through Network Pharmacology, qPCR and CCK-8, we found that baicalein may affect the progression of AD by regulating the expression of PTGS2. Therefore, PTGS2 can be used as a target for AD research, and miR-26a-5p/PTGS2 can be used as an action axis to study the pathogenesis of AD.

## Data Availability Statement

The original contributions presented in the study are included in the article/Supplementary Material, further inquiries can be directed to the corresponding author/s.

## Ethics Statement

The studies involving human participants were reviewed and approved by the Ethics Committee of the Medical Center of the Third Affiliated Hospital of Naval Medical University. The patients/participants provided their written informed consent to participate in this study.

## Author Contributions

TX was responsible for bioinformatics data analysis and writing. YP was responsible for the pharmacologic analysis and experiment of the network, and completes the writing of the results. PS was responsible for cell function experiment operation, data analysis, and result writing. FZ and LH performed the data correction and image rendering. SW designed the study and approved the article for publication. All authors read and approved the final manuscript.

## Conflict of Interest

The authors declare that the research was conducted in the absence of any commercial or financial relationships that could be construed as a potential conflict of interest.

## Publisher’s Note

All claims expressed in this article are solely those of the authors and do not necessarily represent those of their affiliated organizations, or those of the publisher, the editors and the reviewers. Any product that may be evaluated in this article, or claim that may be made by its manufacturer, is not guaranteed or endorsed by the publisher.

## Footnotes

1
https://www.ncbi.nlm.nih.gov/geo/

2
http://sangerbox.com/Tool

3
http://miRWalk.umm.uni-heidelberg.de/

4
https://starbase.sysu.edu.cn/

5
https://david.ncifcrf.gov/

6
http://stringdb.org

7
http://www.gsea-msigdb.org/gsea/downloads.jsp

8
http://herb.ac.cn/

9
http://www.tcmip.cn/ETCM/index.php/Home/

10
https://old.tcmsp-e.com/index.php

## Supplementary Material

Supplementary Material 1 Information of AD patients (20 AD patients and 20 control samples). AD, Alzheimer’s disease.

Supplementary Material 2 AD related Chinese herbal medicine and PTGS2 related Chinese herbal medicine in HERB database and ETCM database. AD, Alzheimer’s disease. Sheet 1: AD related Chinese herbal medicine in HERB database. Sheet 2: PTGS2 related Chinese herbal medicine in HERB database. Sheet 3: AD related Chinese herbal medicine in ETCM database. Sheet 4: PTGS2 related Chinese herbal medicine in ETCM database. AD, Alzheimer’s disease.

Supplementary Material 3 AD related components in HUANGQIN from HERB database. AD, Alzheimer’s disease.

Supplementary Material 4 PTGS2 related components in HUANGQIN from HERB database.

Supplementary Material 5 The main components in HUANGQIN from TCMSP database.

## References

[B1] Ali Syeda Z. Langden S. S. S. Munkhzul C. Lee M. Song S. J. (2020). Regulatory Mechanism of MicroRNA Expression in Cancer. *Int. J. Mol. Sci.* 21:1723. 10.3390/ijms21051723 32138313 PMC7084905

[B2] Alzheimer’s Association (2022). 2022 Alzheimer’s disease facts and figures. *Alzheimer Dement* 18 700–789. 10.1002/alz.12638 35289055

[B3] Ansari A. Maffioletti E. Milanesi E. Marizzoni M. Frisoni G. B. Blin O. (2019). miR-146a and miR-181a are involved in the progression of mild cognitive impairment to Alzheimer’s disease. *Neurobiol. Aging* 82 102–109. 10.1016/j.neurobiolaging.2019.06.005 31437718

[B4] Barbieri I. Kouzarides T. (2020). Role of RNA modifications in cancer. *Nat. Rev. Cancer* 20 303–322. 10.1038/s41568-020-0253-2 32300195

[B5] Barrett T. Wilhite S. E. Ledoux P. Evangelista C. Kim I. F. Tomashevsky M. (2013). NCBI GEO: archive for functional genomics data sets–update. *Nucleic Acids Res.* 41 D991–D995. 10.1093/nar/gks1193 23193258 PMC3531084

[B6] Breijyeh Z. Karaman R. (2020). Comprehensive Review on Alzheimer’s Disease: causes and Treatment. *Molecules* 25:5789. 10.3390/molecules25245789 33302541 PMC7764106

[B7] Busche M. A. Hyman B. T. (2020). Synergy between amyloid-β and tau in Alzheimer’s disease. *Nat. Neurosci.* 23 1183–1193. 10.1038/s41593-020-0687-6 32778792 PMC11831977

[B8] Cai M. Wang Y. W. Xu S. H. Qiao S. Shu Q. F. Du J. Z. (2018). Regulatory effects of the long non-coding RNA RP11-543N12.1 and microRNA-324-3p axis on the neuronal apoptosis induced by the inflammatory reactions of microglia. *Int. J. Mol. Med.* 42 1741–1755. 10.3892/ijmm.2018.3736 29956723

[B9] Chang L. Li K. Guo T. (2017). miR-26a-5p suppresses tumor metastasis by regulating EMT and is associated with prognosis in HCC. *Clin. Transl. Oncol.* 19 695–703. 10.1007/s12094-016-1582-1 27864783

[B10] Congdon E. E. Sigurdsson E. M. (2018). Tau-targeting therapies for Alzheimer disease. *Nat. Rev. Neurol.* 14 399–415. 10.1038/s41582-018-0013-z 29895964 PMC6463489

[B11] de Medeiros L. M. De Bastiani M. A. Rico E. P. Schonhofen P. Pfaffenseller B. Wollenhaupt-Aguiar B. (2019). Cholinergic Differentiation of Human Neuroblastoma SH-SY5Y Cell Line and Its Potential Use as an In vitro Model for Alzheimer’s Disease Studies. *Mol. Neurobiol.* 56 7355–7367. 10.1007/s12035-019-1605-3 31037648

[B12] Dhakal S. Ramsland P. A. Adhikari B. Macreadie I. (2021). Trans-Chalcone Plus Baicalein Synergistically Reduce Intracellular Amyloid Beta (Aβ42) and Protect from Aβ42 Induced Oxidative Damage in Yeast Models of Alzheimer’s Disease. *Int. J. Mol. Sci.* 22:9456. 10.3390/ijms22179456 34502362 PMC8430801

[B13] Di Leva G. Garofalo M. Croce C. M. (2014). MicroRNAs in cancer. *Annu. Rev. Pathol.* 9 287–314. 10.1146/annurev-pathol-012513-104715 24079833 PMC4009396

[B14] Frejborg E. Salo T. Salem A. (2020). Role of Cyclooxygenase-2 in Head and Neck Tumorigenesis. *Int. J. Mol. Sci.* 21:9246. 10.3390/ijms21239246 33287464 PMC7731111

[B15] Hou T.-Y. Zhou Y. Zhu L.-S. Wang X. Pang P. Wang D.-Q. (2020). Correcting abnormalities in miR-124/PTPN1 signaling rescues tau pathology in Alzheimer’s disease. *J. Neurochem.* 154 441–457. 10.1111/jnc.14961 31951013

[B16] Hutter C. Zenklusen J. C. (2018). The Cancer Genome Atlas: creating Lasting Value beyond Its Data. *Cell* 173 283–285. 10.1016/j.cell.2018.03.042 29625045

[B17] Ji Q. Wang X. Cai J. Du X. Sun H. Zhang N. (2019). MiR-22-3p Regulates Amyloid β Deposit in Mice Model of Alzheimer’s Disease by Targeting Mitogen-activated Protein Kinase 14. *Curr. Neurovasc. Res.* 16 473–480. 10.2174/1567202616666191111124516 31713484

[B18] Ji Y. Han J. Lee N. Yoon J.-H. Youn K. Ha H. J. (2020). Neuroprotective Effects of Baicalein, Wogonin, and Oroxylin A on Amyloid Beta-Induced Toxicity via NF-κB/MAPK Pathway Modulation. *Molecules* 25:5087. 10.3390/molecules25215087 33147823 PMC7662334

[B19] Jing C. Sun Z. Xie X. Zhang X. Wu S. Guo K. (2019). Network pharmacology-based identification of the key mechanism of Qinghuo Rougan Formula acting on uveitis. *Biomed. Pharmacother.* 120:109381. 10.1016/j.biopha.2019.109381 31542616

[B20] Kim M. J. Kim J.-H. Kim J. H. Lee S. Cho E. J. (2020). Amelioration effects of *Cirsium japonicum* var. *maackii* extract/fractions on amyloid beta25-35-induced neurotoxicity in SH-SY5Y cells and identification of the main bioactive compound. *Food Funct.* 11 9651–9661. 10.1039/d0fo01041c 33211040

[B21] Liu Y. Wang L. Xie F. Wang X. Hou Y. Wang X. (2020). Overexpression of miR-26a-5p Suppresses Tau Phosphorylation and Aβ Accumulation in the Alzheimer’s Disease Mice by Targeting DYRK1A. *Curr. Neurovasc. Res.* 17 241–248. 10.2174/1567202617666200414142637 32286945

[B22] Miao J. Jing J. Shao Y. Sun H. (2020). MicroRNA-138 promotes neuroblastoma SH-SY5Y cell apoptosis by directly targeting DEK in Alzheimer’s disease cell model. *BMC Neurosci.* 21:33. 10.1186/s12868-020-00579-z 32736520 PMC7393818

[B23] Nagaraju G. P. El-Rayes B. F. (2019). Cyclooxygenase-2 in gastrointestinal malignancies. *Cancer* 125 1221–1227. 10.1002/cncr.32010 30747998

[B24] Noh H. Park C. Park S. Lee Y. S. Cho S. Y. Seo H. (2014). Prediction of miRNA-mRNA associations in Alzheimer’s disease mice using network topology. *BMC Genom.* 15:644. 10.1186/1471-2164-15-644 25086961 PMC4132902

[B25] Prabhakaran J. Molotkov A. Mintz A. Mann J. J. (2021). Progress in PET Imaging of Neuroinflammation Targeting COX-2 Enzyme. *Molecules* 26:3208. 10.3390/molecules26113208 34071951 PMC8198977

[B26] Ramalingam M. Huh Y.-J. Lee Y.-I. (2019). The Impairments of α-Synuclein and Mechanistic Target of Rapamycin in Rotenone-Induced SH-SY5Y Cells and Mice Model of Parkinson’s Disease. *Front. Neurosci.* 13:1028. 10.3389/fnins.2019.01028 31611767 PMC6769080

[B27] Rawat C. Kutum R. Kukal S. Srivastava A. Dahiya U. R. Kushwaha S. (2020). Downregulation of peripheral PTGS2/COX-2 in response to valproate treatment in patients with epilepsy. *Sci. Rep.* 10:2546. 10.1038/s41598-020-59259-x 32054883 PMC7018850

[B28] Serpente M. Fenoglio C. D’Anca M. Arcaro M. Sorrentino F. Visconte C. (2020). MiRNA Profiling in Plasma Neural-Derived Small Extracellular Vesicles from Patients with Alzheimer’s Disease. *Cells* 9:1443. 10.3390/cells9061443 32531989 PMC7349735

[B29] Shi Z. Zhang K. Zhou H. Jiang L. Xie B. Wang R. (2020). Increased miR-34c mediates synaptic deficits by targeting synaptotagmin 1 through ROS-JNK-p53 pathway in Alzheimer’s Disease. *Aging Cell* 19:e13125. 10.1111/acel.13125 32092796 PMC7059146

[B30] Shigemizu D. Mori T. Akiyama S. Higaki S. Watanabe H. Sakurai T. (2020). Identification of potential blood biomarkers for early diagnosis of Alzheimer’s disease through RNA sequencing analysis. *Alzheimer Res. Ther.* 12:87. 10.1186/s13195-020-00654-x 32677993 PMC7367375

[B31] Spence A. D. Trainor J. McMenamin Ú Turkington R. C. McQuaid S. Bingham V. (2019). High PTGS2 expression in post-neoadjuvant chemotherapy-treated oesophageal adenocarcinoma is associated with improved survival: a population-based cohort study. *Histopathology* 74 587–596. 10.1111/his.13786 30408225

[B32] Tan C. Liu L. Liu X. Qi L. Wang W. Zhao G. (2019). Activation of PTGS2/NF-κB signaling pathway enhances radiation resistance of glioma. *Cancer Med.* 8 1175–1185. 10.1002/cam4.1971 30740906 PMC6434213

[B33] Tao W. Yu L. Shu S. Liu Y. Zhuang Z. Xu S. (2021). miR-204-3p/Nox4 Mediates Memory Deficits in a Mouse Model of Alzheimer’s Disease. *Mol. Ther.* 29 396–408. 10.1016/j.ymthe.2020.09.006 32950103 PMC7791017

[B34] Targa A. Dakterzada F. Benítez I. D. de Gonzalo-Calvo D. Moncusí-Moix A. López R. (2020). Circulating MicroRNA Profile Associated with Obstructive Sleep Apnea in Alzheimer’s Disease. *Mol. Neurobiol.* 57 4363–4372. 10.1007/s12035-020-02031-z 32720075

[B35] Tiwari S. Atluri V. Kaushik A. Yndart A. Nair M. (2019). Alzheimer’s disease: pathogenesis, diagnostics, and therapeutics. *Int. J. Nanomed.* 14 5541–5554. 10.2147/IJN.S200490 31410002 PMC6650620

[B36] Wang L. Xu J. Zhan Y. Pei J. (2020). Acupuncture therapy for Alzheimer’s disease: a protocol for an overview of systematic reviews. *Medicine* 9:e20244. 10.1097/MD.0000000000020244 32481301 PMC7249950

[B37] Wang X. Liu D. Huang H.-Z. Wang Z.-H. Hou T.-Y. Yang X. (2018). A Novel MicroRNA-124/PTPN1 Signal Pathway Mediates Synaptic and Memory Deficits in Alzheimer’s Disease. *Biol. Psychiatry* 83 395–405. 10.1016/j.biopsych.2017.07.023 28965984

[B38] Wang Z. Liu T. Xue W. Fang Y. Chen X. Xu L. (2020). ARNTL2 promotes pancreatic ductal adenocarcinoma progression through TGF/BETA pathway and is regulated by miR-26a-5p. *Cell Death Dis.* 11:692. 10.1038/s41419-020-02839-6 32826856 PMC7443143

[B39] Wolin S. L. Maquat L. E. (2019). Cellular RNA surveillance in health and disease. *Science* 366 822–827. 10.1126/science.aax2957 31727827 PMC6938259

[B40] Wu J. Wang B. Li M. Shi Y.-H. Wang C. Kang Y.-G. (2019). Network pharmacology identification of mechanisms of cerebral ischemia injury amelioration by Baicalin and Geniposide. *Eur. J. Pharmacol.* 859:172484. 10.1016/j.ejphar.2019.172484 31229537

[B41] Yoshida K. Yokoi A. Yamamoto Y. Kajiyama H. (2021). ChrXq27.3 miRNA cluster functions in cancer development. *J. Exp. Clin. Cancer Res.* 40:112. 10.1186/s13046-021-01910-0 33766100 PMC7992321

