# Supplementary material for: Identification of miRNA–mRNA Pairs in the Alzheimer’s Disease Expression Profile and Explore the Effect of miR-26a-5p/PTGS2 on Amyloid-β Induced Neurotoxicity in Alzheimer’s Disease Cell Model
Source: Front Aging Neurosci. 2022 Jun 15;14:909222. doi: 10.3389/fnagi.2022.909222 (PMC9249435; doi:10.3389/fnagi.2022.909222)
Supplement: Supplementary file 6 [file Table_1.docx]

Supplementary table 1. MiRNAs and mRNAs in the miRNA-mRNA regulatory network.

| **miRNAs** | **mRNAs** |
| --- | --- |
| hsa-miR-30a-5p | RSBN1，ARHGAP30，SCYL3，RC3H1，KDM5B，DSTYK，ELK4，LPGAT1，AIDA，TP53BP2，LBR，ARID4B，VAMP3，KIF1B，TARDBP，ZBTB40，S100PBP，ZYG11B，LRRC8B，PPP1R12B，MDM4，HLX，MIA3，MPP7，CUL2，ZCCHC24，ATAD1，PLXDC2，BMI1，RET，SLC29A3，DDIT4，SEC23IP，AHNAK，DDX6，LDHA，DGKZ，PAAF1，UBE4A，ARCN1，RNF26，SLC2A3，LMBR1L，RARG，NECAP1，ETNK1，CPSF6，VEZT，ATP2A2，ZNF664，ELF1，IRS2，GMFB，PPP2R5E，SPTLC2，ITPK1，BTBD7，NIPA1，HERC2，MTMR10，UBR1，ZSCAN29，DMXL2，RFX7，TBC1D2B，DET1，TUBGCP5，SNX1，RAB11A，ARIH1，DEXI，SLC38A7，GOT2，ZFHX3，LPCAT2，CTCF，SNTB2，SSH2，ACACA，CBX1，MBTD1，HELZ，FAM104A，PSME3，RPS6KB1，TANC2，NPC1，NAPG，SAFB2，SUGP2，MAST3，ZNF544，SOS1，ASB3，FAM168B，GALNT3，IDH1，MAP4K4，TMEM87B，R3HDM1，WDR75，FKBP1A，STAU1，CABLES2，DIDO1，DLGAP4，PCMTD2，DYRK1A，RRP1B，PITPNB，SLC4A7，CLASP2，LRRFIP2，SEC22C，NAA50，ZBTB20，CCDC14，PIK3R4，DCUN1D1，NR2C2，CAPN7，GNAI2，HPS3，MME，WDR1，UBE2D3，WDR19，TET2，IL15，RAPGEF6，FAM13B，NR3C1，PPP2R2B，SMAD5，RNF14，ARHGAP26，NSD1，TRAM2，ASCC3，PREP，SEC63，REV3L，DSP，CDKAL1，C6orf89，MARCKS，TULP4，YWHAG，STEAP4，ZNF746，ZKSCAN1，ING3，TSPAN33，MKLN1，YWHAZ，KLF10，DERL1，ZHX1，DOCK5，MTDH，B4GALT1，MEGF9，PSMD5，GOLGA1，DENND4C，RAD23B，ZBED1，RPS6KA3，ACSL4，ENOX2，MBNL3，MECP2，ZXDB，ATP7A，STAG2 |
| hsa-miR-26a-5p | NADK，GNB1，UBR4，CAPZB，HP1BP3，SFPQ，KIAA0319L，INPP5B，TMEM9，PPP1R15B，DSTYK，NUCKS1，LPGAT1，EXOC8，B3GALNT2，AHCTF1，VAMP3，UBE4B，KIF1B，TARDBP，PLOD1，ZDHHC18，S100PBP，MACF1，ZYG11B，PDE4B，SRSF11，ACADM，SH3GLB1，HIPK1，RGS4，UCK2，POU2F1，SOAT1，PPP1R12B，ARF1，LARP4B，UPF2，EPC1，ZCCHC24，ATAD1，ZDHHC6，TIAL1，PFKFB3，DHTKD1，ZNF37A，UBE2D1，PCGF5，ZNF518A，FRAT1，NOLC1，TOLLIP，RNF141，FANCF，AHNAK，ATL3，SESN3，BCL9L，PRDM10，QSER1，TRIM44，MED17，FUT4，UBE4A，MLF2，SLC2A3，CKAP4，CORO1C，GIT2，PPTC7，WNK1，NECAP1，ETNK1，RAP1B，TXNRD1，PLBD2，SUDS3，PRKAB1，FOXO1，IRS2，RB1，KLHL28，SOS2，PPP2R5E，ZFP36L1，SYNJ2BP，SPTLC2，ATXN3，BTBD7，PRIMA1，DICER1，KHNYN，SYNE2，MPP5，SLC39A9，BMF，LRRC57，TTBK2，UBR1，GABPB1，MYO5A，RFX7，ZNF280D，TUBGCP5，CYFIP1，ZNF609，RAB11A，FEM1B，IREB2，ZNF592，ABHD2，MCTP2，CREBBP，DNAJA2，AP1G1，FANCA，CENPBD1，NDE1，NFATC2IP，CYLD，NLRC5，KIAA0753，TOM1L2，NUFIP2，SSH2，PHB，MTMR4，MED13，FAM104A，PIPOX，MSL1，WIPF2，NSF，RPS6KB1，TANC2，DCAF7，PPP4R1，PTGS2，ROCK1，MBP，SMAD4，SLC35E1，ARID3A，TPM4，ZNF85，ZNF257，ZNF254，CEBPG，CYTH2，ZNF776，PDIA6，BCL11A，RBMS1，HECW2，TMBIM1，LPIN1，FOSL2，EPAS1，ACTR2，MXD1，MAP4K4，RGPD5，TMEM87B，KIF5C，PLEKHA3，STRADB，PPP1R3D，DIDO1，KIF3B，TTPAL，STK4，RBM38，CBS，SLC5A3，DYRK1A，SF3A1，PISD，MTMR3，NUP50，CRBN，MRPS25，SLC4A7，CLASP2，NEK4，KPNA1，RYK，MAP6D1，SLC6A6，NR2C2，KAT2B，KLHL18，RPP14，BBX，ARAP2，PDS5A，TSPAN5，SLC30A9，REST，PDLIM5，NFKB1，TET2，AIMP1，MMAA，RPS3A，GALNT7，ARSB，CHD1，PPP2R2B，NIPBL，POLK，LNPEP，SMAD5，PURA，SLC36A1，MAT2B，NUP153，FKBP5，SRPK1，EEF1A1，SNX14，SEC63，TSPYL4，FAM50B，MAPK13，PIM1，PHACTR2，STXBP5，TAB2，FBXL18，VOPP1，BCL7B，UBE2H，ZC3HAV1，SLC37A3，PRKAG2，ANKIB1，ZNF655，ZNF277，ING3，NEFL，CCDC25，KIF13B，RBM12B，UQCRB，YWHAZ，SLC25A32，DERL1，B4GALT1，CNTNAP3，ALDH1A1，RFK，SUSD1，ENG，ASB6，NACC2，SEC16A，DENND4C，URM1，HUWE1，ACSL4，AMMECR1，TXLNG，REPS2，DDX3X，OGT，ZBTB33，XIAP，PHF6，SLC9A6 |
| hsa-miR-151a-5p | SLC35E2B，E2F2，SLC25A24，AGTRAP，TNFRSF1B，PKN2，ADAM15，LARP4B，AP3M1，PDZD8，IGF2，TPP1，CORO1B，PITPNM1，BCL9L，RIC8A，TMEM216，KDM2A，UVRAG，ARF3，FRS2，SUDS3，IRS2，PLEK2，SPTLC2，SLC39A9，HERC2，BMF，MYO5A，PARP16，PKMYT1，GOT2，ESRP2，FANCA，TRAF7，RNF40，CAMTA2，MED24，ATXN7L3，MAFG，TAF15，RARA，RPS6KB1，KLF16，RAB3D，SERTAD3，KDELR1，DNM2，CARM1，LENG8，EPN1，CLASP1，PRPF40A，FOSL2，EPAS1，MXD1，RHBDD1，RNF24，PSMF1，STK4，RBM38，SLC19A1，DYRK1A，SLC25A1，PITPNB，TOB2，TTLL12，OSBP2，GRAMD4，SUCLG2，CCDC14，KPNA4，NR2C2，KAT2B，ZNF589，ST6GAL1，ADD1，TBC1D14，AP3B1，KLHL3，WDR36，SLC36A1，XPO5，TULP4，DNAJC30，TMUB1，DBNL，SLC45A4，FBXO25，CHRAC1，ERP44，DENND1A，GOLGA1，ENG，AK1，ZER1，ODF2，EGFL7，PJA1，AMMECR1，MECP2，APEX2，TAZ |
| hsa-miR-101-3p | CRYZ，MCL1，RIT1，RC3H1，KDM5B，LPGAT1，ANGEL2，KIF1B，CDC42，ZBTB40，ZC3H12A，MACF1，SH3GLB1，LRRC8B，AGL，CEPT1，UBAP2L，POU2F1，SOAT1，PPP1R12B，MDM4，RCOR3，MPP7，KIF5B，RRP12，NT5C2，SFXN4，TIAL1，ZMYND11，CSGALNACT2，DDIT4，PLEKHA1，DDX6，ZNF143，QSER1，HIPK3，TRIM44，PPFIA1，PAAF1，UVRAG，ARCN1，ITPR2，AMIGO2，PPTC7，WNK1，ETNK1，SPRYD4，RAP1B，FRS2，ZNF84，ZNF268，PSPC1，ATP11A，RBM23，GMFB，VTI1B，NUMB，SEL1L，RPS6KA5，ATXN3，MPP5，CCNK，WDR20，COPS2，DMXL2，MYO5A，RAB27A，FEM1B，EMP2，CNOT1，AP1G1，ATF7IP2，METTL9，NFATC2IP，ARL2BP，ANKFY1，DHX33，SSH2，SLFN13，ACACA，MMD，MTMR4，MED13，AXIN2，HELZ，MAP2K4，RPS6KB1，TANC2，DCAF7，ZNF235，ZNF714，ZNF257，STRN，TMEM185B，CLASP1，ANKRD44，RHOQ，REL，ACTR2，STAMBP，CNNM4，MAP4K4，RGPD5，NCOA6，ARFGEF2，MOCS3，BACH1，SON，MORC3，DYRK1A，UBP1，PTGS2，GOLGB1，KPNA1，PHC3，ABCC5，KCNH8，BBX，ARMC8，NDUFB5，ARAP2，TSPAN5，UBE2D3，RBPJ，PDLIM5，TET2，IL15，RICTOR，ANKRA2，AP3B1，KLHL3，FAM13B，JAKMIP2，CSF1R，LNPEP，FAM174A，TNFAIP8，CTNNA1，PURA，SLC36A1，EXOC2，ASCC3，REV3L，HIVEP2，SOD2，SRSF3，ZFAND3，NFYA，NT5DC1，ZNF800，ZNF746，USP42，GLCCI1，CCM2，ANKIB1，ZKSCAN1，IMPA1，PLEKHF2，B4GALT1，RNF38，NACC2，TGFBR1，RAD23B，RPS6KA3，REPS2，OGT，DIAPH2，ZBTB33，STAG2，SLC9A6，FHL1 |
| hsa-let-7e-5p | PAFAH2，KIAA0319L，MED8，SERBP1，USP33，TGFBR3，GFI1，DNTTIP2，SLC25A24，GDAP2，GATAD2B，PTGS2，UCHL5，KDM5B，DSTYK，ELK4，LPGAT1，LBR，SIPA1L2，AHCTF1，UBE4B，KIF1B，MACF1，LRRC8B，AGL，PLEKHO1，TOMM40L，POU2F1，SOAT1，PPP1R12B，ATP2B4，ZC3H11A，RCOR3，PITRM1，AP3M1，POLR3A，ZCCHC24，DHTKD1，WDFY4，TOLLIP，NUP98，COPB1，TRAF6，TP53I11，KCTD21，SESN3，BCL9L，HYOU1，FADS2，TM7SF2，ARCN1，CHD4，C1RL，EEA1，PPTC7，ETNK1，SPRYD4，RASSF3，PLXNC1，VEZT，ATP2A2，ZNF268，FOXO1，STK24，PABPC3，UFM1，TRIM13，ATP11A，SNX6，KLHL28，BTBD7，DICER1，TOX4，BCL2L2，CHURC1，TECPR2，MARK3，PACS2，NIPA1，TTBK2，RFX7，CALML4，AP3S2，SNAP23，SERF2，SNX1，ARIH1，ISLR，ABHD2，MAN2A2，CORO7，GLYR1，SLC38A7，ESRP2，METTL9，PHKG2，HEATR3，DDX19B，ZNF276，ANKFY1，PLSCR3，TRAPPC1，PIK3R5，ACACA，MBTD1，MMD，COIL，MTMR4，TRIM37，HELZ，FAM104A，MRPL38，TK1，SGSM2，MINK1，ARHGEF15，MAPK7，GOSR1，RPS6KB1，ROCK1，MBD1，SALL3，ILF3，CARM1，DCAF15，ZNF254，MBOAT2，PREB，SLC5A6，SERTAD2，CLASP1，RBMS1，TRIB2，REL，STAMBP，MTHFD2，MAP4K4，RGPD5，DDX18，PLEKHA3，LRRFIP1，STAU1，HM13，RALY，ARFGEF2，MOCS3，RUNX1，SLC5A3，DYRK1A，TOB2，MTMR3，OSBP2，ADSL，SLC4A7，EOMES，GLB1，TRANK1，SHISA5，IP6K2，NAA50，CD80，ANAPC13，ATR，KPNA4，PHC3，ABCC5，KLHL18，KLHDC8B，PXK，BBX，ADPRH，ARMC8，TTC14，VPS8，UBE2D3，ZNF141，SH3TC1，PDLIM5，TET2，IL15，NUP155，RICTOR，SLC38A9，PDLIM7，PARP8，SMAD5，MATR3，ARHGAP26，NSD1，XPO5，TRAM2，RNGTT，REV3L，HIVEP1，ABCC10，MARCKS，PHACTR2，UTRN，STXBP5，TAB2，ACTB，CDCA7L，OSBPL3，VOPP1，HIP1，COG5，ZC3HAV1，GTF2I，ZNF655，ING3，AHCYL2，MKLN1，BRF2，ADRB3，RBM12B，SLC25A32，FBXO32，SLC45A4，JRK，FAM160B2，DOCK5，PLEKHA2，E2F5，MTDH，RNF139，TLN1，RNF38，GNAQ，TGFBR1，UGCG，OLFML2A，RPS6KA3，MBNL3，MECP2，FLNA，TXLNG，TAZ |
| hsa-miR-15a-5p | GNB1，PAFAH2，PHC2，KIAA0319L，SERBP1，TGFBR3，SLC25A24，RSBN1，BCAS2，ARNT，SELENBP1，GATAD2B，SLC39A1，GBA，ARHGEF11，PEX19，KDM5B，ELK4，TP53BP2，EGLN1，SIPA1L2，ARID4B，AHCTF1，PRKCZ，UBE4B，KIF1B，CDC42，PNRC2，THRAP3，MACF1，ZYG11B，SRSF11，TMEM167B，LIX1L，POU2F1，QSOX1，NPL，LGR6，PPP1R12B，ATP2B4，ZC3H11A，RASSF5，RCOR3，TRAF5，MIA3，TRIM58，MPP7，KIF5B，EPC1，CUL2，DNAJB12，AP3M1，ARL3，NT5C2，UROS，ZMYND11，DHTKD1，PLXDC2，WDFY4，HK1，SGPL1，PLAU，TSPAN14，PCGF5，PI4K2A，GBF1，PLEKHA1，PPP2R2D，TOLLIP，NUP98，TP53I11，KBTBD4，MTCH2，TUT1，BRMS1，MRPL11，ZDHHC24，SPTBN2，INTS4，SYTL2，SESN3，DDX6，BCL9L，C1QTNF5，PRDM10，SNX19，RIC8A，STIM1，SMPD1，HIPK3，NAT10，TRIM44，EXT2，FAM111A，FADS2，ALDH3B1，RELT，PAAF1，EED，PANX1，UBE4A，ARCN1，CACNA2D4，NOP2，CD163，IPO8，TFCP2，CCDC59，EEA1，PPTC7，ETNK1，RASSF3，PLXNC1，VEZT，POLR3B，TCHP，ATP2A2，ZNF664，FOXO1，ATP11A，RBM23，HEATR5A，KLHL28，SOS2，VTI1B，SEL1L，RPS6KA5，DICER1，TOX4，LRP10，BCL2L2，SLC39A9，SIPA1L1，TECPR2，PACS2，NIPA1，MTMR10，LRRC57，UBR1，MYO5A，ZNF280D，CLPX，TBC1D2B，VPS33B，CYFIP1，IVD，SNAP23，SERF2，SNX1，ZNF609，ARIH1，ISLR，ABHD2，MAN2A2，NPRL3，CIAPIN1，SLC38A7，GOT2，RANBP10，VAC14，AP1G1，ZFHX3，FANCA，METTL9，PHKG2，CYLD，C16orf70，SNTB2，MON1B，ANKFY1，CAMTA2，SREBF1，TOM1L2，NUFIP2，ACACA，UBTF，SLC25A39，PHB，MMD，MTMR4，TRIM37，MED13，HELZ，ABCA5，SRP68，TK1，ARHGDIA，SGSM2，MINK1，CYB5D1，MAP2K4，GOSR1，MSL1，STAT5A，RPS6KB1，DCAF7，NUP85，SLC16A3，SMAD7，MBD1，MBP，NAPG，WDR7，CTDP1，PIP5K1C，DPP9，MAN2B1，NR2F6，SUGP2，ZNF626，DYRK1B，KCNN4，BBC3，RDH13，CHMP2A，ILF3，CARM1，SIGLEC7，MBOAT2，ODC1，PDIA6，ROCK2，SLC5A6，REEP1，CLASP1，SAP130，RBMS1，LANCL1，TMBIM1，CAD，FOSL2，GALM，PAPOLG，PEX13，MAP4K4，RGPD5，KIF5C，PLEKHA3，ANKZF1，TBC1D20，RNF24，TRPC4AP，DIDO1，PSMF1，PANK2，CDS2，KIF3B，TTPAL，ARFGEF2，SLC9A8，DNAJC5，RUNX1，SLC19A1，SON，SLC5A3，DYRK1A，COL6A2，PISD，TOB2，PMM1，TTLL12，MTMR3，PRR5，TTC38，TRMU，SLC4A7，UBP1，TRANK1，LRRFIP2，CSRNP1，SHISA5，IP6K2，EIF4E3，ZBTB20，CD80，ANAPC13，KPNA4，PHC3，DCUN1D1，ABCC5，PCYT1A，NR2C2，UBE2E2，KLHL18，ZNF589，ATRIP，KLHDC8B，FLNB，ARMC8，MME，TTC14，ABCF3，VPS8，LRCH3，ARAP2，TSPAN5，UBE2D3，LRBA，SH3BP2，ADD1，TBC1D14，WDR19，PDLIM5，RAP1GDS1，IL15，MMAA，GALNT7，RICTOR，PRKAA1，SLC38A9，TAF7，PCDH1，RPS14，FBXW11，SNX18，PIK3R1，PAM，WDR36，SMAD5，CTNNA1，PURA，PCDHA7，PCDHA5，ARHGAP26，SLC36A1，NSD1，SRPK1，PPIL1，XPO5，TRAM2，SLC17A5，ASCC3，REV3L，SOD2，TCP1，SIRT5，HLA-E，LTA，MAPK14，ABCC10，AIG1，PHACTR2，UTRN，STXBP5，PCMT1，ZFAND2A，INTS1，CDCA7L，OSBPL3，HERPUD2，POLM，VOPP1，HIP1，YWHAG，COG5，UBE2H，PRKAG2，USP42，ANKIB1，GATAD1，ZKSCAN1，PMPCB，AHCYL2，EXOC4，EN2，DNAJB6，STC1，BRF2，NCALD，ZHX1，JRK，PLEC，FAM160B2，DOCK5，FNTA，SDCBP，B4GALT1，TLN1，GBA2，RNF38，GNAQ，SPTLC1，GOLGA1，ASB6，QSOX2，EXD3，DCAF10，TGFBR1，TLR4，ODF2，EGFL7，RPS6KA3，ACSL4，AMMECR1，MBNL3，GPC3，MECP2，FLNA，MPP1，TXLNG，YIPF6，OGT，ATP7A，SLC9A6，TAZ |
