# Supplementary material for: Identification of miRNA–mRNA Pairs in the Alzheimer’s Disease Expression Profile and Explore the Effect of miR-26a-5p/PTGS2 on Amyloid-β Induced Neurotoxicity in Alzheimer’s Disease Cell Model
Source: Front Aging Neurosci. 2022 Jun 15;14:909222. doi: 10.3389/fnagi.2022.909222 (PMC9249435; doi:10.3389/fnagi.2022.909222)
Supplement: Supplementary file 7 [file Table_2.docx]

Supplementary table 2. Overlapping genes between AD characteristic genes and sub-network genes. The italics with underlining indicate the overlapping genes.

|  | **differentially expressed genes** |
| --- | --- |
| **AD characteristic genes** | HERC2, PEX16, TRBV20-1, TRBV6-5, ***TRIB2***, UNC13D, ***PTGS2*** |
| **Sub-network genes** | RPS14, RPS3A, DHX33, DNTTIP2, NOLC1, RRP1B, ***TRIB2***, RRP12, DDX18, WDR75, NAT10, ***PTGS2*** |

AD: Alzheimer's disease.
